# “Summer hypokalemia” as an initial presentation of cystic fibrosis in a morbidly obese African American adult: case report

**DOI:** 10.1186/s12882-020-02130-y

**Published:** 2020-11-07

**Authors:** Yangming Cao, Rachel Donaldson, David Lee

**Affiliations:** 1 Divisions of Nephrology, Department of Internal Medicine, UCSF Fresno Center for Medical Education and Research, 155 N Fresno St, Fresno, CA 93701 USA; 2 The Nephrology Group, 568 E Herndon Ave, Suite 201, Fresno, CA 93720 USA; 3 Divisions of Pulmonology, Department of Internal Medicine, UCSF Fresno Center for Medical Education and Research, 155 N Fresno St, Fresno, CA 93701 USA

**Keywords:** Cystic fibrosis, Hypokalemia, Adult, Obesity, Case report

## Abstract

**Background:**

Most patients with cystic fibrosis (CF) present with respiratory or digestive symptoms. About 3% of patients have electrolyte disturbances at the time of diagnosis, but most of the described cases presenting with this manifestation have been in children. Only 3 adult patients are identified in the literature who first presented with hypokalemia. We describe a morbidly obese African American adult who presented with severe hypokalemia and metabolic alkalosis, which eventually led to the diagnosis of CF after multiple hospitalizations over 4 consecutive summers. Besides being the first African American adult with this presentation, he had the highest BMI, lowest serum potassium, highest pH, and highest bicarbonate level.

**Case presentation:**

In the summer of 2015, a 26 year-old African American man presented to the hospital for generalized weakness. His BMI was 54 kg/M^2^, and he had been on a special diet for a few months with a weight loss of 50 pounds. He sweated profusely while working as a chef. Laboratory tests showed severe hypokalemia and metabolic alkalosis. Further work-up pointed toward extrarenal losses of potassium. He was treated with intravenous normal saline and potassium chloride. After discharge, his potassium level remained normal through the winter while the potassium was tapered off. However, over the following three summers, he repeatedly presented to hospitals for the same problems. Cystic fibrosis was suspected and confirmed by an abnormal pilocarpine sweat test. Gene test revealed two mutations of cystic fibrosis transmembrane conductance regulator (CFTR). Thereafter, his potassium level remained normal with potassium replacement during summertime. Unexpectedly, however, his BMI rose to 83 kg/M^2^ after he stopped the special diet for weight reduction. The reason for the delayed diagnosis is discussed.

**Conclusion:**

We present an exceedingly rare case of CF in a morbidly obese African American adult male whose only manifestation of CF was hypokalemia and metabolic alkalosis. Clinicians should keep an open mind to the diagnosis of CF in ethnically diverse populations, even if it seems unlikely at first glance. For “summer hypokalemia”, consider cystic fibrosis.

## Background

Around 90% of patients with cystic fibrosis (CF) are identified within the first 10 years of life, while about 4% will not be identified until adulthood [1]. CF is caused by cystic fibrosis transmembrane conductance regulator (CFTR) dysfunction and usually presents with respiratory or digestive symptoms. About 3% of patients have electrolyte abnormalities at the time of diagnosis, but most of the described cases presenting with this manifestation have been in children, generally infants [1, 2]. Only 3 adult patients are identified in the English literature who first presented with hypokalemia [2–4]. Due to pancreatic insufficiency and the metabolic demands of chronic lung disease, most patients with CF have a BMI that is below normal [1]. Here, we describe a morbidly obese African American adult man who presented with severe hypokalemia and metabolic alkalosis, which eventually led to the diagnosis of CF after multiple hospitalizations. Our aim is to increase awareness about this rare condition in obese adults, which can be diagnosed if there is a high index of suspicion.

## Case presentation

### First hospitalization

In August 2015, a 26-year-old morbidly obese African American man presented to the hospital with increasingly generalized weakness for 2 months and light-headedness for 4 days. His BMI was 54 kg/M^2^, and he had been on a special diet for a few months with a weight loss of 50 pounds. The diet was mainly composed of vegetables but of unknown detail. He sweated profusely while working as a chef in his own kitchen for a web-based catering service. He had childhood asthma and a pulmonary embolism at age of 21 years. He was born in Hartford, Connecticut to an African American father and a Caucasian mother (a Brazilian immigrant). He moved to Fresno, CA in 2010 and started working for his own catering service in 2015. On exam, his blood pressure was 138/73 mmHg with a pulse of 91 beats/min. His mucous membranes were dry. Laboratory tests showed severe hypokalemia (serum potassium of 1.5 mEq/L) and metabolic alkalosis with prerenal azotemia (Table 1). Arterial blood gas revealed a pH of 7.55, PaCO_2_ of 50 mmHg, PaO_2_ of 60 mmHg and bicarbonate of 42 mEq/L. The urine sodium (< 10 mEq/L) and potassium (13.8 mEq/L) were both very low (Table 1). These findings were consistent with extrarenal losses of potassium. In addition, his 24-h urine aldosterone was normal at 5.4 mcg/d (normal 2.3–21) and free cortisol was also normal. His thyroid function was normal, as were renal ultrasound and renal Doppler ultrasound. After given intravenous normal saline and potassium, he was discharged and prescribed with potassium chloride 20 mEq two tabs bid.

**Table 1:** Our Patient’s Lab Values

Year Setting	Aug 2015 1st Hospital	Apr 2016 Office	Jul 2016 ER	Jul 2017 2nd Hospital	Jul 2018 3rd Hospital
Na (mEq/L)	134	142	136	136	134
K (mEq/L)	1.5	4.3	2.5	2.3	2.7
Cl (mEq/L)	78	101	88	89	91
HCO3 (mEq/L)	42	26	37	38	36
BUN (mg/dL)	15	11	16	17	15
Cr (mg/dL)	1.39	0.75	1.1	1.0	1.1
Urine Na (mEq/L)	< 10			45	26 (FeNa 0.09%)
Urine Cl (mEq/L)	^a^			< 15	
Urine K mEq/L	13.8 ^b^			19	33
Urine Cr mg/dL	215				237
Urine K/Cr mEq/g ^c^	6.4				13.9
Urine pH	5.5			5.0	5.0
Plasma renin (ng/ml//hr) ^d^	28.2 (H)			12.6 (H)	
Plasma aldosterone (ng/dL) ^e^	6 (N)			< 1 (L)	

^a^ Urine Cl was ordered twice but not done; ^b^ K 16.6 mEq/24 h on same specimen (< 30 mEq/24 h indicates extrarenal loss of potassium); ^c^ Urine K/Cr < 22 mEq/g indicates extrarenal loss of potassium; ^d^ Renin normal range 0.25–5.82 ng/ml//hr.; ^e^ Aldosterone normal range 3–16 ng/dL, supine

### Follow-up, emergency room (ER) visit and Second Hospitalization (Table 1)

In follow-up, the patient’s potassium was tapered off. His potassium remained normal through the winter, and so he was discharged from the nephrology office (YC) in April, 2016. His hypokalemia was considered a result of his reduced oral intake while on a special weight loss diet combined with excessive sweating during a Fresno summer. In July 2016, he visited the ER and was found to have a blood pressure of 150/80 mmHg, hypokalemia and metabolic alkalosis. He was given intravenous potassium and discharged the next morning. In July 2017, he was admitted to an outside hospital for the same problems, and another nephrologist was consulted.

### Third hospitalization

In July 2018, he was again admitted for the same complaints (Table 1). Nephrology consultation (YC) was involved. Oral potassium chloride was prescribed at discharge. Because of repeated summertime hypokalemia with excessive sweating, CF was suspected. Initially, the pulmonologist was reluctant to pursue a test for CF because the patient lacked evidence of CF, was morbidly obese, and was from a low incidence demographic. However, further discussion with our CF expert (DL) led to disease specific tests.

### Additional findings and outcome

HRCT showed clear lungs and pulmonary function test was normal. Lipase was low at 8 U/L (normal 12–53). Pilocarpine sweat test demonstrated chloride of 101 mEq/L on the left arm and 106 mEq/L on the right arm (normal < 60). Genetic testing revealed two CFTR mutations: heterozygous p.F508Del (pathogenic mutation) and heterozygous (TG)12-5 T *in trans* conformation, also known as c.[1210–12[5];1210-34TG[12]]. The diagnosis of CF was finally established.

The potassium dose was tapered off over the following 3 months, and the patient’s potassium level remained normal through the winter. In March 2019, his potassium was normal. In anticipation of warmer weather, potassium chloride 20 mEq bid was prescribed. He was advised to moderately increase sodium chloride intake in the summer, but to avoid its excess due to concerns for elevated BP. The monthly lab showed his potassium was maintained at 3.6–4.0 mEeq/L throughout the summer of 2019 while he continued to work in his kitchen. Our long-term plan is to supplement him with potassium from April to October every year. However, his body weight has increased steadily, with a most recent BMI of 87 Kg/M^2^. He is reluctant to consider bariatric surgery because his mother’s surgery was accompanied by complications. He appreciated that we had made a diagnosis and gave informed consent for publication.

## Discussion and conclusion

Based on the consensus guidelines from the Cystic Fibrosis Foundation, the diagnostic criteria of CF rely on both clinical symptoms and evidence of CFTR dysfunction such as an elevated sweat chloride above 60 mEq/L [5]. Our patient has no typical clinical features of CF. The abnormal sweat chloride tests were consistent with CFTR dysfunction. The CF diagnosis was confirmed by the identification of two CFTR mutations. The p.F508Del pathogenic mutation in exon 11 of the CFTR gene is associated with reduced chloride conductance and the typical manifestations of CF [6]. The 5 T variant in intron 9 of the CFTR gene has pathogenic potential as a gene mutation, but low penetrance and gene splicing lead to this gene product being edited out of the final protein [6]. The 5 T variant, in *trans* with a pathogenic CFTR mutation such as p.F508Del or in homozygous state has been associated with CFTR-related disorders such as congenital bilateral absence of the vas deferens, bronchiectasis and acute or recurrent chronic pancreatitis [7, 8]. The length of the poly-TG nucleotide track, which immediately precedes the T track in intron 9 of *CFTR*, influences the penetrance of the 5 T genotype [9]. Our patient is the first African American patient diagnosed with cystic fibrosis with hypokalemia as initial presentation in the adult age.

This case illustrates a highly unusual presentation and delayed diagnosis of CF in a morbidly obese African American adult. Our initial suspicion for CF was minimized because of the following: (1) he had no signs of lung or pancreatic disease; (2) he is African American rather than Caucasian; (3) he was morbidly obese; (4) he was on a special diet for weight reduction; (5) hypokalemia as an initial presentation of CF is very rare in adults; (6) his serum potassium level was consistently normal (though in the winter) while potassium supplementation was tapered off. These considerations resulted in repeated hospitalizations over 4 years with involvement of several different specialists before a final diagnosis was reached. A similar near-miss case would not have been diagnosed if the patient did not suddenly develop abdominal pain immediately before he was discharged from the renal clinic [4].

Dysfunctional CFTR results in excessive chloride and sodium losses in final sweat [10]. When sweat flow rates are high, the sweat chloride can reach 104 mEq/L in CF patients, compared with 36 mEq/L in normal controls [11]. In a warm environment, where sweat rates can reach 2 L/h, CF patients may have massive sweat sodium chloride losses, leading to extracellular fluid (ECF) volume contraction and secondary hyperaldosteronism [3, 10]. In a study comprised of CF infants aged under 7 months, elevated renin and aldosterone were found in the majority of patients [12].

Together with our patient, data on the three adult patients found in the literature are summarized in Table 2 [2–4]. All patients presented during hot weather. They are all males, suggesting their somewhat different roles (e.g., performing more physical labors outdoors) in the society compared with those of females. The urine chloride was all low in every patient. Surprisingly, serum aldosterone was elevated in only one patient while renin was high in only two patients. However, one should be aware that some tests might be delayed by 12–24 h (as in our case) until nephrologists were involved, by which time the patients had already received partial or full volume replacement. In addition to being the only African American, our patient had the highest BMI, lowest serum potassium, highest pH, and highest bicarbonate level.

**Table 2:** Comparison of Case Reports

Author (year)	Bates (1997) [3]	Dave (2005) [2]	Vertolli (2013) [4]	Our patient (2015)
Age (years)	17	36	34	26
Race/Gender	Texan/M	W/M	W/M	AA/M
K (mEq/L)	2.2	2.0	2.2	1.5
HCO3 (mEq/L)	40	38		42
pH	7.49	7.54		7.55
Urine Na (mEq/L)	23 (0.2%) ^a^	< 20	126 ^b^	< 10
Urine Cl (mEq/L)	12 (0.2%) ^a^	< 20	10 ^b^	< 15 ^c^
Urine K (mEq/L)	44 (29%) ^a^	86	5 ^b^	13.8
Plasma renin (ng/ml//hr)	Normal	21.81 (High)	Normal ^b^	28.21 (High)
Plasma aldosterone (ng/dL)	Low to normal	33 (High)	Normal ^b^	6 (Normal)
CFTR Gene Mutation	Heterozygous p. F508Del and R 117H	p. F508Del and 2789 + 2insA	Heterozygous deletion of Exon17a-18 and 2789 + 5 G → A.	Heterozygous p.F508Del and (TG)12-5 T
Other features	3 of 5 siblings with same mutations	Azoospermia, Mild obstruction with air trapping on pulmonary function test, chest HRCT normal and BMI 30.3 kg/M^2^	Azoospermia, ‘tree in bud” pattern and bronchiectasis on CT, and adipose pancreas on CT	Pulmonary function test normal, chest HRCT normal, BMI 54 kg/M^2^ (Recent BMI 83 kg/M^2^ in 2019), Lipase normal

^a^ Percent indicates fractional excretion of each electrolyte, respectively. ^b^ The test was done as outpatient when potassium was partially corrected to 3.2 mEq/L. ^c^ Done during another hospitalization

ECF volume contraction, activation of the renin-angiotensin-aldosterone pathway, and hypokalemia have been proposed to explain the generation and maintenance of metabolic alkalosis [13, 14]. However, our patient did not have elevated aldosterone despite elevated renin. Nonetheless, sweat sodium chloride losses coupled with significant hypokalemia (as in CF) could generate severe alkalosis [15]. In addition, chloride depletion alone has been shown to maintain metabolic alkalosis in rat models and humans, irrespective of volume or potassium status [16]. Pendrin is a luminal Cl^−^/HCO^−−^ exchanger in B intercalated cells along the renal collecting duct [17]. Absence of luminal chloride (as in CF) results in absent bicarbonate excretion from the renal collecting duct (as now there is no luminal chloride for exchange) despite increased pendrin activity during chloride depletion alkalosis. The lack of bicarbonate excretion in the renal tubules helps maintain systemic metabolic alkalosis.

Hyperaldosteronism causes potassium wasting in both the sweat and the urine, resulting in hypokalemia [3]. Our patient mainly had enhanced sweat potassium loss considering his low urine potassium. At high sweat flow rate, the sweat potassium in CF patients can reach 17.8 mEq/L compared with 8.4 mEq/L in normal controls [11]. A CF patient sweating at 2 L/h can lose 178 mEq of potassium over 5 h on a hot summer day, which by itself can cause severe hypokalemia. Metabolic alkalosis can exacerbate urinary potassium losses by increasing distal delivery of sodium bicarbonate, but this did not play a big role in our patient who had low urine potassium and low urine pH.

CF and morbid obesity rarely go hand in hand. The median BMI of patients aged 20–40 years in 2017 was 22.2 [1]. However, because of the early recognition of CF, nutritional supplementation, and increased prevalence of obesity in the general population, obesity within the CF population is not as rare in this decade as in the past. An 8% prevalence of obesity was reported among a pediatric cohort [18]. Nonetheless, morbid obesity remains rare [1]. The prevalence of CF in the Caucasian population is approximately 1 in 3500. In the African American population, however, prevalence is much rarer, affecting 1 in 17,000.

The number of adults being diagnosed with CF is steadily increasing, and this may be due to increased clinical suspicion of the disease [1]. The diagnosis is pursued due to recurrent pulmonary infections, the presence of upper lobe predominant bronchiectasis on chest imaging, malabsorption, and infertility. Associated CF disease modifying mutations are being discovered at an alarming pace. Currently, there are over 1800 gene mutations associated with the diagnosis of CF, although 85% of patients diagnosed with CF harbor at least one copy of the p.F508Del mutation [1]. Due to variable penetrance, the manifestations and severity of disease can vary considerably, and as demonstrated with our case, lead to delayed diagnosis.

In conclusion, we have presented an exceedingly rare case of CF in a morbidly obese African American adult male whose only manifestation of CF was hypokalemia with metabolic alkalosis. Our aim is to further document the heterogeneity of disease manifestation and to encourage clinicians to keep an open mind to the diagnosis of CF in ethnically diverse populations, even if it seems unlikely at first glance. For “summer hypokalemia”, consider cystic fibrosis.

## Data Availability

Not applicable.

## Abbreviations

CF: Cystic fibrosis
CFTR: Cystic fibrosis transmembrane conductance regulator
ECF: Extracellular fluid
